# The State of the Circulatory System in Incurably Deaf Children

**Published:** 1902-01-11

**Authors:** G. Arbour Stephens

**Affiliations:** Hon. Physician to the Royal Cambrian Institution for the Deaf and Dumb


### Hospital Clinics and Medical Progress.

### THE STATE OF THE CIRCULATORY SYSTEM IN INCURABLY DEAF CHILDREN.

By G. Arbour Stephens, M.D., B.S., B.Sc., (Lond.) Hon. Physician to the Royal Cambrian Institution for the Deaf and Dumb.

A striking feature in connection with children in Deaf and Dumb Institutions is that their hands are cold and bluish-looking, at a time when those of other children are comfortably warm. The children at the Royal Cambrian Institution, Swansea, form no exception to the rule. One cannot blame their surroundings for such an unfortunate circumstance. In fact as regards board and lodging, and clothing they are comfortably off.

The Institution buildings are pleasantly situated on the side of a hill, with a S.S.E. aspect. Whenever the sun shines, it shines on them. The sanitation of the place is very good. The children are well fed and clothed; from a hygienic point of view they are much better off than the majority of other children. Yet, notwithstanding all these advantages, they do not appear to be as healthy as other children. The children at the Swansea Institution seem, however, to suffer less from chilblains than those at similar institutions.

Having satisfied myself that external conditions were not sufficient to account for such a difference in health, I decided to make a careful examination of their circulatory systems. The results of this examination, were they given in tabular form, would take up too much space, but an analysis of these tables may provide some interesting reading.

At the Royal Cambrian Institution there are 33 boys and 22 girls. Of the 33 boys, 21, or 63·6 per cent., were born deaf; while out of 22 girls, 12, or 54·5 per cent., are congenital cases. Of the 21 boys who were born deaf, six only had normal hearts, while nine had weak hearts, three had mitral systolic murmurs, and one had a presystolic mitral murmur; the remaining two had each a doubtful presystolic mitral murmur. Of the 12 boys who acquired deafness as the result of scarlet fever, measles, or some other disease, four had normal hearts, three had weak hearts, two a mitral systolic murmur, one a presystolic mitral, one had both a presystolic and systolic mitral murmur, while two others had doubtful mitral murmurs.

Of the 12 girls whose deafness was congenital, four had normal hearts, two weak hearts, two had mitral systolic murmurs, one a presystolic mitral murmur, two had both a presystolic and systolic mitral murmur, and two seemed to have a reduplicated first sound. Of the 10 girls whose deafness was acquired, seven had normal hearts and three had mitral systolic murmurs.

In addition to the above results I noticed that the second sounds in several cases were considerably accentuated over the aortic and pulmonary cartilages.

For purposes of comparison I examined several boys from one of the Swansea schools, but I did not find the second sounds so marked in them as in the deaf children. Previous to this systematic examination I had occasion to attend the seventh boy on the list, of which the above is an analysis, for a fainting attack. Examination at that time revealed the fact that his heart was slightly irregular, and the first sound blurred. I ordered him tincture of digitalis with the result that the fainting attacks ceased, and as the tables show at the subsequent examination, the heart improved. In several cases the heart sounds were so weak that I was unable to decide definitely whether the heart was really normal or not. The pulse in the majority of cases was regular, but very weak. In a few cases the pulse was irregular; in one it was very rapid, in another very slow (60 per minute), while in yet another it was markedly dicrotic. Several of the children seem rather small for their age, two have badly-shaped chests, one has anchylosis of the jaw, and one has tubercular disease of the wrist. These results show that incurably deaf children, in addition to their inability to hear, are not as healthy as other children. They require special treatment, both mentally and physically. They ought not to be allowed to go in for much vigorous exercise. Properly regulated exercises should form part of the school's curriculum. These exercises should be of such a nature as gradually to develop the muscles without any undue strain upon the heart. Deaf children, unless watched, are particularly liable to fall during gymnastic exercises. If a suitable series of exercises could be decided on the physical condition of the children would be greatly improved, and, as a result, the intellectual condition would be much benefited.

In comparing the work done by deaf children with that done by their more healthy contemporaries, considerable allowance should be made not only for their lack of hearing, but for their weaker physical condition as well. This remark is especially applicable to those gentlemen who hold the position of Inspectors of Schools, and who, passing from the ordinary Board School to a Deaf Institution are inclined to treat the deaf children in the same way as they do the others. In fact, the Inspectors of deaf children should be chosen from among those who have lived and taught in Deaf Institutions. The physical condition of the children has a marked bearing on the selection of a calling for them in after life. No child should be put into any trade that requires great exertion unless he has been medically examined and pronounced fit. We thus see that deaf children are very considerably handicapped compared with their more fortunate brothers and sisters, both as regards school work and the choice of a calling in adult life. A question that naturally arises is, what is the connection between incurable deafness and heart disease—

*a*. In children born deaf;

*b*. In children who have acquired deafness.

As regards the former class, development is so dependent on a good circulatory system that anything which interferes with the proper working of the heart, necessarily affects the development of such an elaborate apparatus as the ear. A weak heart is unable to furnish an adequate supply of pure blood to the developing ear of the fœtus. This inability is still more marked in cases where there is regurgitation or obstruction at the mitral orifice. Can anything be done during the period of gestation to improve or aid the fœtal heart especially in cases where the family history of deafness is very pronounced ?

In children who have acquired deafness the disease which has produced the deafness has most probably affected the heart. The important question is—which is attacked first, the heart or the ear. If the heart is the first to become affected, we can understand how, the driving power being diminished, the circulation is too feeble to remove the local cause of the inflammation in the ear. If the ear is the first to be attacked and then the heart, the connection between the two is not so apparent. Assuming that the former is the correct view, that the heart is the first to fail, our course of treatment is fairly well marked. In scarlet fever especially, and in measles and other fevers to a less extent, greater attention ought to be paid to the circulation. In scarlet fever the extreme rapidity of the pulse must have a very weakening effect on the heart, which in some cases becomes unequal to its task. We ought, therefore, to anticipate such an unfortunate result. We should so direct our treatment as to reduce the frequency of the pulse and thus indirectly, as well as by more direct means, strengthen the heart.

